# The Path to Loneliness for Psychiatric Patients: A Qualitative Study of a Journey Marked by Pain, Hopelessness, Prosocial Signaling Deficits, and Coping Strategies That Are Not Effective

**DOI:** 10.1111/sjop.13089

**Published:** 2025-01-17

**Authors:** Rebecca Landquist, Caisa Öster, Martina Isaksson, Martina Wolf‐Arehult

**Affiliations:** ^1^ Psychiatry Northwest, Region Stockholm Sollentuna Sweden; ^2^ Department of Neuroscience, Psychiatry Uppsala University Uppsala Sweden; ^3^ Centre for Psychiatry Research, Department of Clinical Neuroscience Karolinska Institutet and Stockholm City County Stockholm Sweden

**Keywords:** coping skills, loneliness, prosocial signaling, psychiatric patients, qualitative study, thematic analysis

## Abstract

Enduring loneliness has serious physical and mental health implications. Patients with mental health problems are at risk of experiencing problems related to loneliness. Therefore, it is important to increase knowledge about how loneliness is experienced and managed in this particular group. The aim of the study was to explore (1) psychiatric patients' experiences of different forms of loneliness, (2) associated problems, including difficulties with prosocial signaling, and (3) strategies used to combat loneliness, to better understand how loneliness affects psychiatric patients and how patients manage their loneliness. A total of 110 psychiatric patients were recruited at eight outpatient clinics in Region Stockholm for a larger study of loneliness. The first fifteen patients who also agreed to participate in the present substudy were invited to meet a trainee psychologist who conducted a semi‐structured interview. A reflexive thematic analysis with a codebook approach was used to analyze the transcripts. The described experiences of loneliness were primarily examples of social and emotional loneliness with one prominent theme: “Hopelessly lonely”. Associated problems were summarized in two themes: “The inevitable road to loneliness” and “Social signals are confusing and push others away”. Regarding patients' strategies for combating loneliness, one theme emerged: “Using strategies that focus on the current moment”. The results also included a total of sixteen subthemes. Loneliness was described as something painful that is inevitable and unchangeable, with a self‐reinforcing loneliness loop leading to social and emotional loneliness, and as something that is intertwined with mental health problems. These results are in accordance with research. In addition, patients described a variety of prosocial signaling deficits and feelings of being disconnected from others. They also reported using strategies that primarily alleviated their immediate suffering when they were alone, rather than focusing on approaches with long‐term effects on reducing loneliness, such as participating in social activities combined with effective social signaling. Future research should investigate whether increased awareness of social signaling, as well as social activities combined with improved prosocial signaling and strengthened self‐belief, would constitute effective steps for patients to combat enduring loneliness. It also seems important to help patients reduce hopelessness related to loneliness.

**Summary**

- Results from qualitative interviews showed that psychiatric patients experience loneliness as painful, inevitable and unchangeable, intertwined with their mental health problems and linked to a self‐reinforcing loneliness loop and hopelessness.
- Social signals are experienced as confusing rather than helpful when patients try to connect with others.
- Patients’ strategies to leave loneliness are ineffective, primarily used to alleviate their immediate suffering when they are alone, rather than focusing on approaches with long‐term effects on reducing loneliness, such as participating in social activities combined with effective social signalling.

**Abbreviations**
CBT: cognitive behavioral therapy
DJGLS: De Jong Gierveld Loneliness Scale
M.I.N.I.: Mini International Neuropsychiatric Interview

## 1 Background

The experience of loneliness is subjective, and may differ between different people and situations, but it is always related to a discrepancy between a preferred and an actual level of connectedness, and results in an aversive emotional reaction (Cacioppo et al. 2015). Loneliness can be a result of, for example, the person's personality style (Vanderbleek and Gilbert 2018), learning history (de Heer et al. 2022), earlier experiences and life events (Hensley et al. 2012), or ability to develop close relationships (Arnold and Winkielman 2021). In addition, social coordinates such as a lack of easily accessible meeting places in public spaces may increase the risk of loneliness (Birken et al. 2023). In some situations, the lack of connectedness is intense because the person is alone or without access to the preferred number of social relations and social network (social loneliness), but people also experience a lack of connectedness while being amid family or friends (emotional loneliness) (Russell et al. 1984). In this case, the preferred level of closeness and the feeling of being loved and accepted are lacking. Some people also describe an experience of non‐connectedness with the universe and meaninglessness (Bolmsjö, Tengland, and Rämgård 2019). This is called existential loneliness and overlaps with existential dilemmas, such as the fact that we cannot fully understand each other because each individual life experience is unique. It has also been suggested that the concept of loneliness can be divided into different dimensions, such as intimate, relational, and collective loneliness (Cacioppo et al. 2015). Intimate loneliness refers to the perceived absence of a significant other, relational loneliness refers to the absence of quality friendships or family connections, while collective loneliness refers to the absence of a social identity such as belonging to a sports team, a volunteer group, or other types of groups in society (Cacioppo et al. 2015). Recent studies have shown that loneliness is a prevalent problem in society, and that awareness of its serious consequences increased in the aftermath of the dramatic Covid‐19 lockdowns (Hickin et al. 2021).

People who experience a severe and enduring form of loneliness are often stuck in a self‐reinforcing feedback loop, including negative expectations and judgmental thoughts, a high degree of suspicion, hypersensitivity to social exclusion, and an activated threat system in social situations which trigger social behaviors and signals that keep others at a distance (Hawkley and Cacioppo 2010). Severe and enduring loneliness also correlates with unhealthy lifestyle habits (Kobayashi and Steptoe 2018) and has serious physical health implications (Leigh‐Hunt et al. 2017). Moreover, loneliness correlates with depression (Mushtaq et al. 2014), borderline personality disorder (Liebke et al. 2017), suicidal ideation (Rudatsikira et al. 2007), schizophrenia (Suman et al. 2023), psychosis (Badcock et al. 2015), and autism (Hymas, Badcock, and Milne 2022). Accordingly, the prevalence of loneliness is higher in psychiatric populations compared to non‐psychiatric populations (Beutel et al. 2017), and it has been reported that psychiatric problems can lead to social isolation and an increased risk of loneliness, while loneliness can increase mental health problems (Rokach 2014). Specifically, it has been suggested that prosocial signaling deficits can contribute to loneliness in certain patient groups (Hempel, Vanderbleek, and Lynch 2018). The concept of prosocial signaling entails the non‐verbal communication between individuals, aimed at resolving conflicts and facilitating social bonds (Hempel, Vanderbleek, and Lynch 2018). In a broader sense, however, all behaviors that are conducted in the presence of other people can function as a social signal, influencing interpersonal relationships and our ability to connect (Lynch 2018).

Despite these serious implications of loneliness, there is a lack of guidelines on how to detect and support patients in psychiatric care who suffer from loneliness. There are also no guidelines on how healthcare professionals should take loneliness into account when treating psychiatric disorders, assuming that loneliness could affect patients' ability to respond to a psychotherapeutic intervention (Hickin et al. 2021).

A recent review and meta‐analysis (Pearce et al. 2023) stated that methodological and statistical heterogeneity is a reoccurring problem in research on social isolation and loneliness, as is a lack of longitudinal studies and alternatives to cross‐sectional studies, preventing us from understanding outcomes and mechanisms that increase and maintain loneliness. Another review and meta‐analysis reported that psychological interventions for loneliness are effective, and that cognitive behavioral therapy (CBT) is the most commonly implemented treatment (Hickin et al. 2021). Many interventions, however, are not originally designed to target loneliness, or are not intended for psychiatric patients. Moreover, interventions do not take into account that lonely people constitute a heterogeneous group, and we know little about the mechanisms of change and the long‐term benefits of these interventions (Hickin et al. 2021). Interestingly, Käll et al. (2021) have presented a personalized use of a modular approach and the use of a cognitive behavioral analysis to better understand the patient's path to loneliness. Radically open dialectical behavior therapy (RO DBT) represents another approach to target loneliness associated with excessive overcontrol and mental health problems by, for example, analyzing and improving prosocial signaling (Lynch 2018). Nevertheless, we need to understand more about how different forms of loneliness affect psychiatric patients and which strategies help them to know what components treatments and local support services should include, and which support intervention or treatment works best for which patient in mental healthcare settings, rather than assuming that one size fits all (Hickin et al. 2021). One way to achieve this is to investigate psychiatric patients' experiences in this area with qualitative interviews (Pearce et al. 2023).

Qualitative research has the potential to provide additional insights into a topic such as loneliness through feedback from people who experience it first‐hand. Braun and Clarke (2013) emphasizes that a qualitative study explores multiple realities, creates an in‐depth understanding of a phenomenon, commits to the participants' viewpoints, and disrupts the natural context at a minimum. Results from qualitative studies of loneliness in psychiatric patients have, for example, shown that patients with a borderline personality disorder describe constant feelings of loneliness and disconnection from self and others, together with feelings of numbness and nothingness that can lead to serious maladaptive coping methods such as self‐harm, even though some effective strategies such as behavioral activation and distraction were also reported (Miller, Townsend, and Grenyer 2021). Similarly, adults with autism spectrum disorders report that negative feelings associated with loneliness are persistent (Umagami et al. 2022). Having relationships with shared interests, participating in social activities, improving social skills, and demonstrating positive views and acceptance of oneself can decrease loneliness in autism, whereas anxiety, depression, a lack of understanding, and stigma can increase it. Another recent study reports that stigma and self‐stigma have been associated with mental health problems, and can be a barrier to developing a sense of belonging (Birken et al. 2023). It has also been reported that the ability to connect with others can be improved in psychiatric patients by learning effective prosocial signaling (Isaksson et al. 2021), and that the inability to connect with others and mental health appear intertwined in a way that we need to explore further (Birken et al. 2023). To our knowledge, there has been no qualitative evaluation of different forms of loneliness together with associated problems such as prosocial signaling deficits in psychiatric patients.

The aim of the study was to explore (1) psychiatric patients' experiences of different forms of loneliness, (2) associated problems, including difficulties with prosocial signaling, and (3) strategies used to combat loneliness. This in‐depth exploration can help us understand how loneliness affects psychiatric patients and how patients manage their loneliness, which could also shed light on which components treatments and support services could benefit from including.

## 2 Methods

### 2.1 Research Design

This interview study was part of a larger study of loneliness. A reflexive thematic analysis (Braun and Clarke 2021b) was used to identify, analyze, and report patterns of meaning in data. Ethical approval was obtained from the Swedish Ethical Review Authority (Ref. no. 2021‐05392‐01).

### 2.2 Participants, Setting, and Procedure

A total of 110 patients were recruited from eight outpatient clinics in Region Stockholm for a larger study of loneliness. The clinics offer treatment for all psychiatric patients in need of highly specialized psychiatric care except for severe eating disorders and severe substance use disorders, as these patient groups are treated elsewhere in Stockholm. Information about the study was posted in the outpatient units, providing patients with contact details for the psychologist in charge of recruitment, giving them the opportunity to obtain further information about the study. The psychologist contacted patients who showed an interest in the study by telephone to provide information about the larger study of loneliness, and to assess inclusion and exclusion criteria. All patients were asked to participate in an investigation of the relationship between loneliness and other variables using self‐report questionnaires. Initially, and until fifteen patients had been recruited, patients were also asked to participate in a qualitative interview about loneliness. A total of five patients either declined or withdrew from the offer. The interviewees met the inclusion criteria of a minimum age of 18 years and currently receiving outpatient care at the clinic, and none of the exclusion criteria. The exclusion criteria included being unable to fill out questionnaires independently due to severe mental illness, i.e., when patients needed inpatient care or other emergency interventions, or due to insufficient cognitive abilities or knowledge of Swedish. In accordance with the guidelines in Region Stockholm, all patients had been assessed with a Mini International Neuropsychiatric Interview (M.I.N.I.) (Lecrubier et al. 1997) after referral. The last registered diagnosis by a medical doctor was reported as the primary diagnosis (see Table 1). All patients who wanted to participate received verbal and written information about the interview study, and gave their written consent via a digital platform.

**TABLE 1:** Descriptive data for study patients.

Descriptive data (*n* = 15)	
Mean age (standard deviation)	41.4 years (SD = 12.47, range 20–64)
Gender	
Male	5 (33.3%)
Female	10 (66.7%)
Marital status	
Married or in a relationship	4 (26.7%)
Single	11 (73.3%)
Highest level of education completed	
Elementary school	3 (20%)
High school	8 (53.3%)
University	4 (26.7%)
Main occupation	
Working	5 (33.3%)
Studying	3 (20%)
Unemployed	2 (13.3%)
Sick leave	5 (33.3%)
Mean level of loneliness (DJGLS)	9.4 (SD = 2.5, range 2–11)
Diagnosis	
Attention‐deficit/hyperactivity disorder	4 (26.7%)
Autism	3 (20%)
Depression	5 (33.3%)
Bipolar disorder	1 (6.7%)
Anxiety syndrome	1 (6.7%)
Number of comorbid diagnoses	1.67 (range 1–3)

To describe the level of loneliness in the sample, loneliness was assessed with the De Jong Gierveld Loneliness Scale (DJGLS) (see Table 1), which is an 11‐item self‐rated questionnaire (De Jong‐Gierveld and Van Tilburg 2006). The scale has been validated in several European, Asian, and American countries, and the correlation between the DJGLS and another well‐known instrument is high (*r* = 0.88, *p* < 0.01) (Caycho‐Rodríguez et al. 2023). Responses were dichotomized and a sum score was calculated resulting in a value between 0 and 11 for each patient, with a higher score indicating a higher level of loneliness (De Jong‐Gierveld and Van Tilburg 2006). Descriptive data for study patients is presented in Table 1.

### 2.3 The Interviews

A semi‐structured interview guide was developed to explore the patients' experiences of loneliness, with three question areas derived from the aim. It began with an open question asking participants to describe their experiences of loneliness, with follow‐up questions regarding the experiences of social, emotional, and existential loneliness, including a brief explanation of each concept. Social and emotional loneliness were included because these aspects of loneliness are relatively easy for patients to understand, are often mentioned in discussions about loneliness in society today, and are also included in widely used self‐rated instruments that measure loneliness such as the DJGLS. Existential loneliness was included because of its potential importance to healthcare staff and psychiatric patients, with the assumption that existential issues might arise when confronted with serious illnesses and psychiatric symptoms related to meaning of life, such as depression and suicidality. Subsequently, the patients were asked to describe problems associated with loneliness, including possible difficulties with prosocial signaling. Prosocial signaling as a concept was briefly explained and one or two examples were presented, such as seeking eye contact and showing a soft and friendly smile (Lynch 2018). Finally, the patients were asked to describe which strategies can be helpful to combat loneliness (strategies with negative or positive consequences). Probing questions were used throughout each interview to help the patients develop their answers or give examples. The duration of the interviews ranged from 11 to 55 min.

Prior to the interviews, participants were informed that the interviews would be anonymized with identifying details removed before analysis. A date was set for the interview, which was conducted by a trainee psychologist between December 2022 and February 2023. The psychologist who performed the interviews was not otherwise involved in the study, and did not already know the patients. The interviews were audio‐recorded, and most of them were transcribed verbatim by another trainee psychologist who was not otherwise involved in this study. Three of the interviews were transcribed by the first author. Details that could lead to the patient or the interviewer being identified were removed.

### 2.4 Data Analysis

The reflexive thematic analysis emphasizes the researcher's subjectivity as a resource through his or her reflexive engagement with theory, data, and interpretation. The importance of each data point is determined by its ability to capture something important in relation to the research question. In this study, a codebook approach was used which represents a combination of coding reliability approaches and reflexive approaches—a codebook is created from the data, and the data is coded into this codebook (Braun and Clarke 2021a).

To ensure methodological quality, all authors were involved in the analyses. Transcripts were reviewed separately by the first, second, and last authors, who also conducted the thematic analyses. All authors participated in reviewing the codebooks and final analyses. The second author has extensive experience in conducting qualitative analyses.

First, the interviews were read and reread to give the reviewers familiarity with the contents. Second, the text was coded within three a priori themes emanating from the question areas in the interview guide: Experiences of loneliness (social, emotional, and existential loneliness), associated problems, including difficulties with prosocial signaling, and strategies that have been helpful (with negative or positive consequences) to combat loneliness. Possibilities to find additional themes were considered. The a priori themes were then used as main themes. Third, themes and subthemes were generated within the main themes by identifying patterns in the text that were relevant for the research question and aim. To make sure the themes and subthemes represented the data, the interviews were reread. Fourth, the first and the last authors compared their findings with the second author, discussed and resolved any differences, and came up with preliminary names for the themes. Fifth, the process of reviewing the themes, going back to the interviews, recoding under the new themes, and discussing was performed a second time. Lastly, codebooks and themes with codes were presented to the third author and disagreements were discussed until agreement was achieved.

## 3 Results

The analysis rendered four themes with subthemes, sorted into the three main themes, as follows: (1) Hopelessly lonely, (2) The inevitable road to loneliness, (3) Social signals are confusing and push others away, and (4) Using strategies that focus on the current moment. Quotations are used to illustrate findings (see Table 2).

**TABLE 2:** Main themes, themes, subthemes, and quotations.

***Main theme: Experiences of different forms of loneliness (social, emotional, and existential loneliness)***	
**1. Theme: “Hopelessly lonely”**	
**Subtheme**	**Quotations**
1.1 No one is there for me	– *My friends don't want to hang out with me*. – *There is no one to turn to*. – *Others don't understand me*.
1.2 I feel pain	*When I am lonely:* – *… it is painful*. – *… my feelings of emptiness are extreme*, *and everything seems hopeless*. – *… it really hurts and it crushes my self‐esteem*.
1.3 A longing for closeness and friends	– *I long for someone so much that my body hurts*. – *I just miss being able to chat*, *hang out*, *and do things*. – *It's to do with not having friends*. *My brother doesn't really count*, *because he's family*.
1.4 A downward spiral of hopelessness	– *It becomes a downward spiral of depression and darkness*, *a feeling that no one cares*, *and then you become more depressed*. – *I am stuck in a negative loop… I kind of don't deserve them*, *it is a punishment for withdrawing all these years [because of mental health problems]*, *and now I'm going to die alone and it doesn't feel good*. – *No one else wants to join you… It's no fun to do things on your own… and then there is a lot of self‐criticism… when you feel that nobody likes you*. – *I get so lonely and it feels like it is pointless to try [to change things]*.
***Main theme: Problems associated with loneliness*, *including difficulties with prosocial signaling***	
**2. Theme: “The inevitable road to loneliness”**	
**Subtheme**	**Quotations**
2.1 My mental health problems and my loneliness are intertwined	– *I was depressed for a long time… I buried myself at home and eventually people stopped calling*, *and I understand that*. – *When I am not doing well*, *I feel alone too*, *and get anxious and feel bad*. – *I know that loneliness is not a psychiatric disorder*, *but loneliness can become difficult and having psychological difficulties – it makes me lonely*.
2.2 Circumstances make socializing difficult	– *I realized… that my best friends were married or lived abroad*, *so they didn't have the time to meet in the same way as before*. – *It is not easy to keep friends after a difficult divorce*. – *When I moved… it became difficult to visit friends at home*. – *Where I live*, *people sometimes… judge you… and are not interested in getting to know you if they have a negative impression*. – *As a grown‐up*, *it is more difficult*, *everybody has their group that they belong to already*.
2.3 Loneliness just happened to me	– *Friends stopped calling me*, *I don't know why*. – *I always come up with suggestions for what we can do*. *No one else takes the initiative… I give up*. – *I was a really social child*, *but now I just can't do it anymore*. – *It has always been difficult to find friends*.
2.4 I see loneliness as my own fault	– *This situation is my own fault*. *It is possible to register for a course or something [to meet others]…* – *I feel that it is like this because of me*. – *Why am I not good enough?*
**3. Theme: “Social signals are confusing and push others away”**	
**Subtheme**	**Quotations**
3.1 I feel different and social signaling confuses me	– *My needs don't match my friends' needs*. – *I think that others think I am weird and I don't understand these social codes*. – *I have to think about what I say and should not say all the time… Do they think I am too much?* – *There's no point in trying to make a connection… If they knew what I think about [thoughts about mental health problems]*, *they wouldn't think I'm any fun to talk to*. – *Wherever I go*, *people don't like me*. – *[If somebody is friendly] I have a hard time understanding if the person I am talking to is really looking for a friend or just trying to be nice*.
3.2 I lack strategies to socialize, and my signals may push others away	– *All the sensory input [in social situations] is difficult*, *so I sit silently*. – *This thing about not being able to express yourself and explain to people how you feel*. *I became very alone in difficult situations*, *since I couldn't reach out to people*. – *Small talk isn't something I am used to doing*. – *Meeting new people is difficult*, *I don't even know where to start*. *When we have talked about the weather*, *then what?* – *(Due to my mental health problems) I have almost forgotten how to socialize*. *I didn't really dare to open up… and become close to the others*. – *If I leave a party*, *it is because I feel that I have no one to talk to… and then I ask myself*, *why did I do that?* – *When I feel bad… and someone talks to me*, *I just look in another direction*. – *I don't even check other people's signals when I am preoccupied with not doing well or trying to look happy*. – *I have often been told that I look angry*, *even though I'm not*. – *When you are not feeling well and your energy is low*, *I think that there is no interest in connecting with others*.
3.3 When I feel safe and secure, I am caring and genuine with friends	– *We always talk a lot*, *and I can trust him and he trusts me*. – *With this person*, *I could be the person I am without having to be ashamed or anxious*. – *You help each other and you are there without demanding anything in return*, *you do it because you want to*.
***Main theme: Strategies to combat loneliness (effective as well as ineffective)***	
**4. Theme: “Using strategies that focus on the current moment”**	
**Subtheme**	**Quotations**
4.1 Participating in social activities and behavioral activation	– *I go to the store to talk a little bit with people*. – *I have found connectedness with people I meet at social services who*, *like myself*, *have sat alone at home too much*. – *When I want to connect with others*, *I go online*, *and chat or play games with others*. *It's real for me*. – *I get positive energy from meeting my family*, *even if I also get tired of socializing*. – *I take my dogs out for a walk*. *That helps*.
4.2 Focusing on positive activities, distracting from loneliness, or self‐soothing	– *I watch movies or take a walk in the forest*, *so I don't feel alone*. – *I like to paint and craft*, *be creative*, *and distract myself from loneliness*. – *I constantly keep myself distracted at home to avoid thinking about how I am not talking to anyone*. – *At home*, *I snuggle up with blankets and a hot cup of tea to feel safe and take extra care of myself*.
4.3 Accepting the moment	– *I don't really know how to describe this*, *but I simply accept the loneliness*, *that it is present*.
4.4 Avoiding the hardship of social situations	– *In social situations*, *I try to avoid attention as much as possible*. *I avoid eye contact*. – *It's taboo*. *I don't talk about my situation (to make things easier)*. – *If I don't feel well enough*, *I just leave [the social situation] and watch TV instead*. – *I talk to people online and don't dare to meet them face‐to‐face*. – *I have become a passive observer (in social situations)*. – *I drink alcohol or take pills to handle anxiety*. *Alcohol makes it feel good in the current moment*, *but it's not worth it*.
4.5 Desperately seeking contact	– *I ask a question because one should talk*, *not because I am really interested in the answer*. *I fake it*, *and it doesn't feel good*. – *I stay in relationships even though I would feel better if I left them*. *It's a kind of safety behavior*. – *When someone gets close*, *you desperately hold onto that person and think: “Oh*, *don't leave me*, *please!”* – *I initiate sexual contact to get close*, *but sometimes these men are not good for me*. – *I adapt too much*, *and in the long run it will hurt me if I don't notice how I am really doing in the relationship*.

### 3.1 Main Theme: Experiences of Different Forms of Loneliness (Social, Emotional, and Existential Loneliness)

#### 3.1.1 Theme 1: “Hopelessly Lonely”

No one is there for me.

A variety of situations that trigger loneliness were described. The examples of social loneliness were related to having no one to meet or talk to, and being alone when longing for social contact, as well as having difficulty finding something to talk about when participants actually have the opportunity for social contact. The examples of emotional loneliness entailed difficulty feeling close to family or friends and having no one to share inner thoughts and problems in life with, while longing for feelings of being understood and loved. Initially, there were few direct examples of existential loneliness. Later during the interviews, however, feelings of meaninglessness and hopelessness were described due to existential dilemmas, such as the inability to fully share a unique experience with others, such as suffering caused by loneliness.

I feel pain.

Loneliness was described as leading to strong aversive feelings and reactions, characterized by intense suffering. In addition, there was a constant feeling of hopelessness and self‐esteem was reduced when repeatedly feeling lonely.

A longing for closeness and friends.

Loneliness was described as a result of an unfulfilled need for connectedness and closeness. Friendship was of great importance, as not everyone has a partner or family relationships, and partnership or family relationships may not be sufficient. The longing could become even more intense if there was already an experience of close relationships earlier in life.

A downward spiral of hopelessness.

Enduring loneliness can lead to a loop of negative thoughts, aversive feelings, and unhelpful behaviors, with a tendency to self‐criticize, become depressed, passive, and withdrawn, and spend a lot of time focusing on negativity and distrust. This loop continues as a downward spiral, with feelings of not being loved or not being able to turn the situation around.

### 3.2 Main Theme: Problems Associated With Loneliness, Including Difficulties With Prosocial Signaling

#### 3.2.1 Theme 2: “The Inevitable Road to Loneliness”

My mental health problems and my loneliness are intertwined.

An interplay between loneliness and mental health problems was noted, for example calls from friends stopped after a while, when a depression‐induced isolation had been going on for a long time, i.e. mental health problems that led to isolation and loneliness. Anxiety and depressive moods could also be triggered by difficulties in social interactions and building closeness, while social interaction became more difficult to handle when participants were anxious and stressed.

Circumstances make socializing difficult.

External circumstances sometimes prevented socializing. For example, moving to a new city or abroad created barriers to staying in close contact with existing friends. Other people's marriages, which limited the time available for social activities, the experience of a difficult divorce, or a demanding work situation could make it more difficult to keep friendships flourishing. Building friendships as an adult was seen as demanding, as it more often than not involves the need to be accepted by the members of a pre‐existing group. Sometimes other people's prejudices also created barriers that were difficult to overcome.

Loneliness just happened to me.

Difficulties understanding what had led to loneliness existed; friends suddenly stopped calling, there was a lack of reciprocity in taking the initiative from the beginning, resulting in the relationship coming to a halt, or there was trouble finding people with similarities, without understanding why.

I see loneliness as my own fault.

Blaming oneself when stuck in loneliness was common. In particular, there was a tendency to not feel good enough, as well as strong self‐criticism, such as not trying hard enough when, for example, choosing not to enroll in a course—a missed opportunity to make friends.

### 3.3 Theme 3: “Social Signals Are Confusing and Push Others Away”

I feel disconnected from others and social signaling confuses me.

A feeling of being disconnected from others was described, where lonely persons experienced that they were different in terms of both personality and needs. A feeling of not being liked by others was also present. One hypothesis was that the mental health problems could be a reason for being rejected. It was described as being difficult to pay attention to and interpret social signals, leading to an experience of this part of the interaction as complex and confusing. In some cases, a constant mental battle was described which involved debating whether or not something was acceptable to say out loud, or whether or not others were genuinely interested.

I lack strategies to socialize, and my signals may push others away.

Socializing was described as difficult, for example knowing how to initiate conversations or explain one's feelings to others. A lack of practice in social settings exacerbated these problems, but a high level of distress could also result in an inability to read other people's social signals. When in distress, facial expressions were described as possibly disadvantageous, such as looking angry despite being interested in social contact and wanting to be friendly.

When I feel safe and secure, I am caring and genuine with friends.

The safety and security of having an unconditional relationship was highlighted, and how this created a welcoming and friendly environment. When feeling safe and secure with a genuine friend, the anxiety and shame disappeared, and it was suddenly possible to interact, as an atmosphere of trust and a mutual willingness to help was built.

### 3.4 Main Theme: Strategies to Combat Loneliness (Effective as Well as Ineffective)

#### 3.4.1 Theme 4: “Using Strategies That Focus on the Current Moment”

Participating in social activities and behavioral activation.

Social contexts in everyday life were sought, for example by visiting stores, meeting people at social services, chatting on the internet, and visiting family members. These activities were part of a behavioral activation regimen to reduce loneliness, sometimes including spending time among people. Taking the dog for a walk as a method of reducing the level of loneliness was also mentioned.

Focusing on positive activities, distracting from loneliness, or self‐soothing.

Activities, such as watching an interesting movie, going for walks, painting, crafting, or drinking one's favorite tea surrounded by soft blankets were described. These activities distracted from the feeling of loneliness, and aimed to focus on other things, increase positive emotions, or self‐soothe. Being constantly distracted was actually described as a way of avoiding the fact that there were no conversations with others going on.

Accepting the moment.

Accepting the presence of loneliness without doing anything more about it was, in some way, a relief.

Avoiding the hardship of social situations.

Social situations could be complicated and result in negative feelings, often leading participants to seek strategies to avoid such complexity altogether. Talking to others online instead of face‐to‐face and avoiding attracting attention while in social situations were methods used to manage aversive feelings. Drugs, alcohol, or behaviors such as leaving parties early in favor of watching TV alone at home were also used to avoid the hardships of social situations. In addition, negative feelings often caused participants to avoid social situations altogether.

Desperately seeking contact.

Negative assumptions about other people were ignored, and time and energy were put into keeping company with those people, even if it was unwanted. A fear of being alone led to a clinginess to other people and to prioritizing other people's needs. Initiating sexual contact was also mentioned as a strategy to avoid loneliness. All of these examples were connected to negative feelings, but despite these emotional reactions, the behaviors could not be stopped.

## 4 Discussion

This study is the first to provide a qualitative evaluation of psychiatric patients' experiences of different forms of loneliness together with associated problems, including difficulties with prosocial signaling. It also investigates strategies to combat loneliness. Loneliness was described as painful, leading to sadness and emptiness, and with a constant presence of hopelessness. This is not surprising, since research has shown that social exclusion leads to strong aversive reactions (Hartgerink et al. 2015) and that loneliness is associated with depression (Ward et al. 2023), emptiness (Miller, Townsend, and Grenyer 2021), and hopelessness (Niu et al. 2020). This result is also in line with a recent qualitative study in which lonely adults with mental health problems described emotional pain when they did not feel understood and loved, and when they did not have their emotional needs met (Birken et al. 2023).

Participants emphasized the importance of having access to different types of relationships—someone to spend time with and conduct activities with to avoid social loneliness, as well as someone who is understanding and accepting, and with whom they feel close, to avoid emotional loneliness. In general, patients gave plenty of examples of both social and emotional loneliness, while examples of existential loneliness were few (van Tilburg 2021). It is likely that existential loneliness remains abstract and more difficult to relate to when asked directly about this experience, and that patients need help during an interview to reflect on how it affects them. In accordance with this, research has suggested that existential loneliness is a multi‐faceted concept with different possible interpretations (Álvarez et al. 2023). Nevertheless, many patients referred to existential dilemmas later on in the interviews, for example by raising questions about the meaning of life, related to the fact that they feel lonely all the time, asking questions about who they are, related to the inability to connect with others, or raising questions about the possibility to fully understand each other, related to the fact that each experience is unique, such as the pain of being lonely. Interestingly, it has been suggested that emotional loneliness most closely represents what people in a non‐psychiatric sample mean when they report loneliness (van Tilburg 2021).

A longing for connectedness and closeness among the participants was expected, since this is seen as a basic human need (Pavey, Greitemeyer, and Sparks 2011), and is important for both our mental and cognitive health (Haslam et al. 2015). Although a close relationship with a brother, sister, father, or mother is valuable and meaningful, as is a close relationship with a partner or one's own child, several patients described a longing for a meaningful relationship and closeness with someone outside the immediate family to feel less isolated and lonely. Friendships protect people against mental and physical illness, and make people feel happier and more content with life (Dunbar 2018).

A complex interaction between thoughts, feelings, and behaviors was described that result in a self‐reinforcing loneliness loop involving hostility, pessimism, stress, anxiety, unhealthy lifestyle habits, and low self‐esteem. This downward spiral that leads to and reinforces loneliness has been described many times by researchers (Hawkley and Cacioppo 2010). It was also suggested that mental health problems are strongly intertwined with loneliness. This is supported by reports that the prevalence of loneliness is higher among psychiatric populations compared to non‐psychiatric populations, and that loneliness has been shown to correlate with different psychiatric disorders, as mentioned in the background. A bi‐directional link was also reported in the qualitative study by Birken et al. (2023).

Participants often believed that they were unable to change or overcome the circumstances that caused their loneliness, such as losing friends after a difficult divorce. Sometimes they did not even understand why they were stuck in loneliness, or they simply concluded that they themselves were to blame. Thus, the road to loneliness may seem inevitable. Indeed, longitudinal results suggest that external and uncontrollable causal beliefs predict less social participation and greater loneliness (Newall et al. 2009). In addition, lonely people have been described as hypersensitive to social exclusion, reacting with higher levels of negative emotion than people who are not lonely, and as hyposensitive to social inclusion, reacting with lower levels of enthusiasm, as well as attributing social inclusion to circumstantial factors and social exclusion to internal and stable characteristics (Vanhalst et al. 2015). However, because DJGLS scores indicate a high level of loneliness in this study sample, these participants may have a particularly difficult time describing and finding solutions to their loneliness.

Social signals were described as confusing, rather than helpful, and participants seemed to be unaware of the possibility of practicing prosocial signaling to improve their chances of building connectedness. It is unclear whether cognitive functioning influenced the ability to describe and understand social signaling, given that 20% of patients reported having only completed elementary school. Nevertheless, difficulties understanding social signaling were reported, at least to some extent, regardless of level of education. For future studies, it seems important to help patients identify and describe their social signals due to the difficulty of grasping the concept of social signaling, for instance by providing multiple descriptions or video recordings of social signals. It would also be possible to ask patients to participate in role plays in which they demonstrate their social signals during social activities, in order to fully capture patients' use of social signals and the impact of these signals on others.

As a result of prosocial signaling deficits, social signals were instead described as pushing others away, despite a longing for closeness. Some of the included participants in a meta‐synthesis by Ikhtabi et al. (2022) expressed a strong desire to connect with others but found it hard investing in relationships or understanding others' intentions. These findings are important because it illustrates a deeper understanding of patients' difficulties with getting stuck in loneliness and the problems that occur during social activities, as well as new possibilities to combat loneliness. Several research teams have already shown that this is a meaningful target for patients with depression (Gilbert et al. 2023), autism (Cornwall et al. 2021), and anorexia nervosa (Isaksson et al. 2021). An improvement in prosocial signaling could reduce the negative effects of the rejection sensitivity related to loneliness (Gao et al. 2017). Despite the difficulties with prosocial signaling, participants also described an ability to be warm and caring with friends and family when they felt safe and secure. Accordingly, it has been suggested that the activation of our body's safety system is an important prerequisite for prosocial signaling (Lynch 2018).

In this study, various strategies to combat loneliness were described by patients. Some are known to be effective, such as behavioral, social, and physical activation (Franke et al. 2021; Käll et al. 2021), while others are known to be ineffective, or even destructive, such as consuming alcohol to reduce anxiety or acting in desperate ways to initiate and maintain contact with others—behaviors that can damage both the relationship and the person's self‐respect. Avoidance behavior, such as staying at home instead of engaging in social activities, was also described as a frequent problem. Part of this result may be explained by the overlap between social anxiety and loneliness (Maes et al. 2019). Some of the effective strategies mentioned in the interviews are examples of skills included in dialectical behavior therapy, such as distraction through activities or cognitive tasks, self‐soothing, accepting the moment to improve distress tolerance, or emotion regulation skills to increase positive emotions (Linehan 2014). A major problem, however, is that many of these strategies focus on the present moment to find short‐term relief, rather than aiming to bring about long‐term changes to reduce loneliness. In particular, patients often performed their strategies alone, although it would have been possible for them to try to include others in these activities or to choose another activity that others could be part of. This pattern of behavior further increases loneliness. Overcoming loneliness requires a combination of several skills, such as being aware of prosocial signaling and being open to participating in social activities with an activated safety system. Such a safety system facilitates the use of effective social signals to connect with others, and creates a positive experience that motivates further contacts for both patients and the people they are engaging with.

### 4.1 Strengths and Limitations

The main limitation of this study was the inclusion of patients who had already agreed to participate in a study focusing on loneliness. This subgroup of patients may be particularly interested in loneliness or may suffer from loneliness to a greater extent and may therefore differ from psychiatric patients in general. However, this was the first qualitative study to investigate different forms of loneliness and prosocial signaling, and it was therefore important to deepen the understanding of loneliness by interviewing people who experience it firsthand.

As the sample size is quite small, the interpretation of the results should be cautious. However, as the data seemed to overlap to some extent, the sample size could be considered sufficient. The aim was not to generalize but rather to provide a richer understanding of some aspects of the experience of loneliness in psychiatric patients, which made the qualitative method suitable.

Another limitation could be that there was not a focus on the experience of loneliness within a specific psychiatric diagnosis. Even though it is important to explore loneliness in specific diagnostic patient groups, our aim was to find common ground among psychiatric patients in general. This is important when developing transdiagnostic educational and treatment programs.

The first and the last authors were responsible for the recruitment and the development of the interview questions, and they also participated in the qualitative analyses. This might have affected how the data was interpreted. To address this issue and to create a diverse research group, two experienced researchers without any involvement in the study and with extensive knowledge of qualitative research and psychotherapy were invited to perform the analyses.

## 5 Conclusions and Future Research

Patients described social and emotional loneliness as painful and intertwined with their mental health problems and as the result of a downward spiral of hopelessness that is difficult to break. The results showed that most patients find it difficult to describe and understand social signaling—something that can contribute to patients getting stuck in a negative self‐reinforcing feedback loop that leads to behaviors that keep others at a distance. Future research should investigate whether increased awareness of social signaling, as well as increased social activities combined with improved prosocial signaling and strengthened self‐belief, would constitute effective steps for patients to combat enduring loneliness. It also seems important to find ways to reduce patients' hopelessness related to loneliness and to develop interviews that are more suitable for investigating existential loneliness.

## Author Contributions

All authors contributed substantially to each part of the manuscript. The first draft of the manuscript was written by Rebecca Landquist and Martina Wolf‐Arehult. All authors read and approved the final manuscript.

## Ethics Statement

This study was performed in line with the principles of the Declaration of Helsinki. Ethical approval was obtained by the Regional Ethics Committee in Stockholm (Ref. no. 2021‐05392‐01). Informed consent was obtained from all individual participants included in the study.

## Consent

Patients signed informed consent regarding publishing their data.

## Conflicts of Interest

The authors declare no conflicts of interest.

## Data Availability

Data available on request due to privacy/ethical restrictions.
